# Lipid Metabolic Effects Induced by Individual and Combined Exposure to Multiple Food Additives in Human Cells

**DOI:** 10.3390/toxics14060487

**Published:** 2026-06-03

**Authors:** Zizhao Huang, Weichunbai Zhang, Xudong Jia, Hui Yang, Ling Yong, Jin Fang, Yan Song, Yi Wan

**Affiliations:** 1 Food Safety and Health Research Center, School of Public Health, Southern Medical University, Guangzhou 510515, China; zizhao308734672@163.com; 2 NHC Key Lab of Food Safety Risk Assessment, China National Center for Food Safety Risk Assessment, Beijing 100021, China; zhangweichunbai@cfsa.net.cn (W.Z.); jiaxudong@cfsa.net.cn (X.J.); yanghui@cfsa.net.cn (H.Y.); yongling@cfsa.net.cn (L.Y.); 3 Laboratory for Earth Surface Processes, College of Urban and Environmental Sciences, Peking University, Beijing 100871, China; wany@urban.pku.edu.cn

**Keywords:** food additive, combined exposure, lipid metabolism, food safety evaluation

## Abstract

With the widespread coexistence of multiple food additives, their combined exposure has raised increasing health concerns. This study used high-content imaging to investigate the individual and combined effects of five common food additives on lipid metabolism in HepG2, Caco-2, and Jurkat T cells. In HepG2 and Caco-2 cells, all additives dose-dependently induced lipid and free cholesterol accumulation; by contrast, Jurkat T cells exhibited only sporadic statistical differences without biologically relevant dose-dependent effects. Notably, lipid droplet accumulation appeared as a relatively early and readily detectable response, though its suitability as a definitive biomarker of metabolic disturbance warrants further validation. Combined-exposure experiments, conducted in HepG2 and Caco-2 cells, revealed cell-specific interaction patterns: additive interactions dominated in HepG2 cells, while sodium benzoate and sodium cyclamate showed synergistic disruption in Caco-2 cells. These results provide in vitro evidence that multi-component combined exposure causes cell-specific lipid-related perturbations not fully captured by single-additive assessment, suggesting that mixture toxicity models should be incorporated to improve food safety evaluation.

## 1. Introduction

Food additives serve as indispensable components of modern food industries, playing irreplaceable roles in improving sensory qualities and extending shelf life. However, with the continuous growth in global consumption of ultra-processed foods, complex additive systems—including sweeteners (such as aspartame, cyclamate, and saccharin sodium), preservatives (sodium benzoate), and colorants (tartrazine)—are frequently incorporated into various food products. Taking non-alcoholic beverages as an example, these additives are commonly used in combination, creating realistic scenarios where consumers are inevitably exposed to multiple additives simultaneously through daily dietary intake [1,2,3,4,5,6,7]. This reality not only challenges traditional food safety risk assessment and regulatory frameworks centered on single components but has also garnered widespread public attention alongside rising health consciousness and occasional food safety incidents [8].

In recent years, accumulating epidemiological and experimental evidence has uncovered potential health hazards linked to three major categories of commonly used food additives: sweeteners, preservatives, and colorants.

As representative artificial sweeteners, aspartame, cyclamate, and saccharin sodium have attracted substantial toxicological concerns. In 2023, the International Agency for Research on Cancer (IARC) classified aspartame as a Group 2B carcinogen (possibly carcinogenic to humans) [9]. Experimental studies have demonstrated that aspartame not only significantly increases liver cancer risk in experimental animals but may also induce male reproductive toxicity, manifested as pituitary-testicular axis hormone disruption, testicular oxidative stress, and apoptosis [10,11,12]. Early animal experiments suggested that saccharin sodium and cyclamate were associated with bladder cancer in rats at extremely high doses [13,14,15]. Furthermore, large-scale prospective cohort studies in Europe and the United States have confirmed associations between artificial sweetener consumption and elevated risks of breast cancer, obesity-related cancers, and cardiovascular diseases [16,17]. Recent research has also revealed that, compared with non-consumers, individuals consuming ≥1 serving per day of sugar-sweetened or artificially sweetened beverages exhibited significantly increased risks of metabolic dysfunction-associated steatotic liver disease, cirrhosis, and chronic liver disease mortality, with proteomic signatures revealing potential biological mechanisms involving inflammation and metabolic dysregulation [18].

As a widely used preservative, sodium benzoate also presents multiple potential adverse biological effects. The preservative sodium benzoate can react with ascorbic acid in beverages under specific conditions (such as light exposure and high temperature) to generate trace amounts of benzene (classified by IARC as a Group 1 carcinogen). In vitro studies have indicated that sodium benzoate can induce DNA damage, micronucleus formation, and chromosomal aberrations in human lymphocytes, and exert potential adverse effects on immune function, hepatic and renal metabolism, and reproductive health through oxidative stress pathways [19,20,21]. Some studies have also suggested associations between its intake and attention deficit hyperactivity disorder (ADHD) risk in children [22,23].

The common colorant tartrazine possesses evident biological safety risks as well. The colorant tartrazine can trigger non-IgE-mediated allergic-like reactions in sensitive individuals and is significantly associated with behavioral disorders in children, including hyperactivity, irritability, and sleep disturbances. Animal experiments have also suggested potential toxicity to the nervous system and liver function [24,25,26,27,28]. This risk evidence for individual food additives indicates that combined exposure to multiple additives may trigger more complex health hazards, highlighting the necessity and urgency of targeted combined toxicity studies.

The new toxicological paradigm centered on New Approach Methodologies (NAMs)—through deep integration of human cell-based models, organ-on-a-chip systems, and computational toxicology while strictly adhering to the 3R principles (Replacement, Reduction, and Refinement of animal experiments)—has transcended the inherent limitations of traditional single-component assessments [29,30,31]. Nevertheless, two critical gaps persist: no unified, standardized in vitro evaluation framework has been established globally to systematically assess combined exposure effects of multi-component food additives; and current systems remain unable to quantify complex chemical interactions, including synergistic, additive, or antagonistic effects. These limitations underscore the urgent need for human-relevant models to inform cumulative risk assessment of additive mixtures [32,33,34]. More importantly, systematic studies on interactions among multiple food additives remain limited, with current research predominantly relying on traditional animal experiments that face ethical constraints, high costs, and uncertainty in extrapolation to human exposure scenarios [35]. The U.S. EPA has explicitly proposed the goal of phasing out mammalian testing by 2035, further highlighting the urgent need for human-relevant in vitro models [36].

In this study, we conducted a comparative analysis of the effects of five frequently co-occurring food additives—including three sweeteners (aspartame, cyclamate, and saccharin sodium), the preservative sodium benzoate, and the colorant tartrazine—across diverse human cell models, including HepG2 cells, Caco-2 cells, and Jurkat T cells. Furthermore, through the examination of lipid metabolism-related indicators using high-content imaging technology, we ascertained the effects of individual and combined exposure to these additives. Isobologram analysis was further employed to elucidate interactions among different additive combinations. These results may provide scientific evidence to facilitate a comprehensive health risk assessment of combined food additive exposure.

## 2. Materials and Methods

### 2.1. Chemicals and Reagents

Aspartame (CAS# 22839-47-0, Cat# PHR1381, Pharmaceutical Secondary Standard, purity 99.7% (HPLC)), sodium benzoate (CAS# 532-32-1, Cat# PHR1231, Pharmaceutical Secondary Standard, purity ≥ 99.0% (HPLC)), and saccharin sodium (CAS# 128-44-9, Cat# PHR1348, Pharmaceutical Secondary Standard, purity ≥ 99.0% (HPLC)) were purchased from Sigma-Aldrich (St. Louis, MO, USA). Cyclamic acid sodium (CAS# 7139-05-9, Cat# S109885, analytical standard, purity ≥ 99.5% (HPLC)) and tartrazine (CAS# 1934-21-0, Cat# T102072, analytical standard, purity ≥ 99.0% (HPLC)) were obtained from Aladdin Biochemical Technology Co., Ltd. (Shanghai, China). The detailed information for reagents used in this study is provided in Supplementary Table S1.

### 2.2. Cell Culture

Human T lymphocyte cell line Jurkat T cells were purchased from ATCC (Manassas, VA, USA). The cells were maintained in RPMI 1640 medium with 10% (*v*/*v*) FBS and 1% (*v*/*v*) penicillin-streptomycin (P/S). HepG2 cells derived from human liver hepatocellular carcinoma were sourced from the Cell Bank of Type Culture Collection Committee of the Chinese Academy of Sciences and maintained in EMEM medium with 10% (*v*/*v*) FBS and 1% (*v*/*v*) P/S. Caco-2 cells derived from Cell Resource Center Institute of Basic Medical Sciences, CAMS/PUMC. The cells were maintained in DMEM medium with 20% (*v*/*v*) FBS and 1% (*v*/*v*) penicillin-streptomycin (P/S). All cells were maintained at 37°C in a humidified atmosphere containing 5% CO_2_, and 95% humidity.

### 2.3. Cell Treatment

Adherent cells (HepG2 and Caco-2) were seeded in 96-well plates at densities of 1.5–3.0 × 10^5^ cells/well for HepG2 and 1.0–2.0 × 10^5^ cells/well for Caco-2, with a volume of 100 μL per well. After incubation at 37 °C with 5% CO_2_ for 24 h, the cells were exposed to test compound solutions prepared by two-fold serial dilution for 48 h. Suspended cells (Jurkat T) were cultured in 48-well plates. Cells were harvested by centrifugation, counted, and then plated at 1.0 × 10^6^ cells/well with a volume of 500 μL per well. Following exposure to test compound solutions (two-fold serial dilution) for 48 h, the cells were incubated with working solutions of PMA (32 μM) and Ionomycin (0.52 μM) at 2.5 μL per well (1:400 dilution) for 12 h to induce activation according to a previous study [37]. Vehicle controls were included in all experiments, and each treatment concentration was performed in triplicate wells. All experiments were conducted with solvent controls. In each independent experiment, at least 2 replicate wells were set for each concentration, and all data were obtained from at least 3 independent experiments.

### 2.4. Cell Staining and High-Content Imaging

High-Content Imaging (HCI) was performed as previously described [38,39]. After 48 h of treatment, cells were fixed with 4% paraformaldehyde (PFA) for 15 min at room temperature, followed by washing with PBS. The cells were then incubated with Filipin III (1:200 dilution) and a lipid droplet dye (1:2000 dilution), as well as a nuclear dye 7-AAD (1:100 dilution), for 30 min at room temperature in the dark. The cells were washed twice with PBS and resuspended in PBS.

Fluorescent images were acquired using an ImageXpress Micro Confocal High-Content Imaging System (Molecular Devices, LLC., 3860 N First Street, San Jose, CA 95134, USA, version 6.5) at 20× magnification, with nine non-overlapping fields captured per well and unified exposure times applied across all samples (DAPI: 300 ms; FITC: 40 ms; Texas Red: 250 ms). Image segmentation and object identification were performed using MetaXpress software (Molecular Devices, version 6.5), with fluorescence thresholding calibrated using blank controls and unified via the Otsu automatic thresholding method (grayscale 120–255); to ensure analytical consistency, nuclei, lipid droplets, and free cholesterol–positive signals were defined based on specific morphological criteria, namely nuclei as circular or oval objects ranging from 50 to 200 μm^2^, lipid droplets as cytoplasmic objects 0.5–5 μm in diameter, and free cholesterol as diffuse or granular cytoplasmic fluorescence. Subsequent image analysis was conducted using CellReporterXpress Imaging and Analysis Software (Molecular Devices, LLC., 3860 N First Street, San Jose, CA 95134, USA, version 6.5), where the expression levels of target proteins were quantified as the mean stain area (MSA), defined as the average stained area per cell (in μm^2^) across all cells in each image and calculated by normalizing the total fluorescent staining area and corresponding object counts to the total cell number in the same image.

Notably, the present HCI data only reflect alterations in lipid droplet accumulation and free cholesterol staining patterns, which are interpreted as lipid-related phenotypic changes. This evidence does not directly demonstrate changes in lipid metabolic flux, de novo synthesis, oxidation or transport, and relevant interpretations are therefore stated with appropriate caution.

### 2.5. Benchmark Dose (BMD_10_) Estimation

The benchmark dose (BMD) modeling approach was used to analyze the concentration-effect relationships of food additives. BMD analysis was performed using the European Food Safety Authority (EFSA) web-tool based on the R-package PROAST (Version 70.0, Dutch National Institute for Public Health and the Environment, RIVM, The Netherlands) as previously described [40]. Model fitting followed EFSA default procedures, and final BMD estimates were derived via weighted averaging of four dose–response models (Exponential, Hill, Inverse Exponential, Log-Normal); 95% confidence intervals (BMDL/BMDU) were calculated from 200 bootstrap resampling iterations. The benchmark response (BMR) was defined as a 10% effect level in dose–response for food additive-induced lipid droplet accumulation and free cholesterol accumulation as visualized by LD staining and Filipin staining.

The isobologram analysis, first proposed by Fraser in 1870 and later improved by Loewe in 1926, is a classical statistical method for evaluating combined effects [41]. In this study, the obtained BMD_10_ values and their corresponding 95% lower and upper confidence limits (BMDL_10_ and BMDU_10_) were combined with the isobologram approach following the procedure described by Wang [39] to analyze the interaction types of combined food additives. Briefly, a two-dimensional coordinate system was constructed with BMD_10_ of two combined additives as X and Y axes, and theoretical additive isoboles were plotted for BMDL and BMDU. Synergism was defined as measured points inside the BMDL isobole, additivity as between the two isoboles, and antagonism as outside the BMDL isobole.

### 2.6. Cytotoxicity Evaluation Method

Cytotoxicity was explicitly assessed based on total cell number quantified by 7-AAD nuclear staining via High-Content Imaging (HCI); no significant reduction in cell count relative to the control group was defined as the absence of overt cytotoxicity, ensuring that observed lipid-related phenotypic changes were independent of cytotoxic effects.

### 2.7. Statistical Analysis

All data were analyzed and graphed using GraphPad Prism (version 10.0). Data conforming to a normal distribution were expressed as the mean ± standard deviation (Mean ± SD), while non-normally distributed data were presented as the median (interquartile range, IQR). Comparisons between two or multiple groups were conducted using the unpaired two-tailed *t*-test, one-way or two-way analysis of variance (ANOVA), followed by the Bonferroni post hoc test for pairwise comparisons. For data with unequal variances, the Welch’s *t*-test (*t*’-test) or Kruskal–Wallis test was applied. A *p*-value < 0.05 was considered statistically significant, and all experiments were independently repeated in triplicate.

## 3. Results

### 3.1. Effects of Individual Food Additives on Lipid Metabolism in Multiple Cell Models

#### 3.1.1. Effects of Individual Food Additives on Lipid Metabolism in HepG2 Cells

Five treatment groups were established (1, 0.5, 0.25, 0.125, and 0.0625 mM) plus a solvent control. The selection of this concentration range was grounded in human biomonitoring data and clinical pharmacokinetic studies. These five food additives have been widely detected in human plasma, with estimated physiological concentrations below 0.0056–0.14 mM, calculated using a 60 kg adult model based on ADI values, 2–5% oral absorption, and 3L plasma volume [42]. Clinical data further support this range: Endo et al. reported safe benzoic acid peaks at 234 μg/mL (~1.67 mM) during clinical treatment [43], while Zhang et al. detected urinary saccharin at 8100 ng/mL (~0.044 mM) [44]. Biomonitoring studies have reported comparable detection levels in human blood and urine samples for the remaining additives [44,45,46,47]. Given that in vitro cell experiments involve substantially shorter exposure durations than real-world human exposure and cannot fully recapitulate in vivo cumulative metabolic effects, higher treatment concentrations were incorporated to ensure detectable biological responses. Accordingly, 1 mM was selected as the maximum in vitro concentration. Furthermore, preliminary experiments confirmed the absence of cytotoxicity at 1 mM (Figure S1), validating the appropriateness of this upper limit. This concentration remains below the clinically reported safe peak of benzoic acid while avoiding non-physiological cytotoxicity.

**Figure 1:**
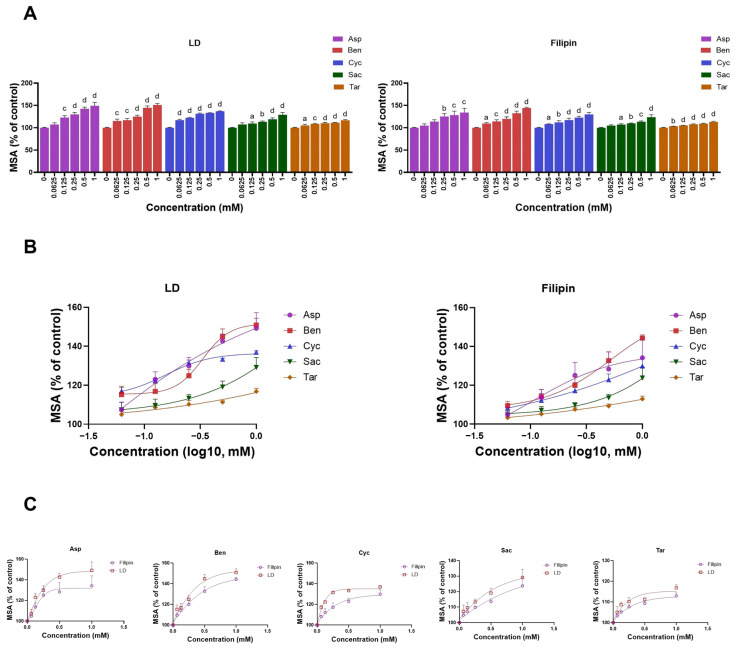
Effects of aspartame (Asp), sodium benzoate (Ben), cyclamate (Cyc), saccharin sodium (Sac) and tartrazine (Tar) on free cholesterol accumulation (Filipin) and lipid accumulation (LD) in HepG2 cells. (**A**) The effect of five additives on the average dyeing area of Filipin and LD (histogram). (**B**,**C**) Dose-effect curves of five additives on Filipin and LD; Unpaired t test or one-way analysis of variance (one-way ANOVA) were used for significance test. a, *p* < 0.05; b, *p* < 0.01; c, *p* < 0.001; d, *p* < 0.0001; MSA (% of control): the percentage of the average staining area of the treatment group to the average staining area of the control group; The error bar represents the mean ± standard deviation of three biological replicates.

The results showed that none of the treatment concentrations exhibited cytotoxicity (Figure S1A). All food additives displayed a clear dose–response relationship and significantly induced free cholesterol accumulation (Filipin staining, *p* < 0.001) and lipid accumulation (LD staining, *p* < 0.001) in HepG2 cells (Figure 1A,B), with lipid accumulation showing a greater increase than free cholesterol accumulation(Figure 1C). Among the five food additives tested, aspartame, sodium benzoate, and sodium cyclamate induced accumulation effects more potently than sodium saccharin and tartrazine (Figure 1B). Cell imaging analysis revealed that, at the 1 mM treatment concentration, all five food additives significantly increased the mean staining areas (MSA) of free cholesterol (Filipin) and lipids (LD) compared with the control group (Figure S2). These findings demonstrate that the five food additives trigger dose-dependent aberrant phenotypic changes in cholesterol membrane distribution and intracellular lipid accumulation in HepG2 cells, indicating that food additive exposure can alter hepatic lipid-related phenotypic profiles, which may potentially impair normal hepatocyte physiological function.

#### 3.1.2. Effects of Individual Food Additives on Lipid Metabolism in Caco-2 Cells

**Figure 2:**
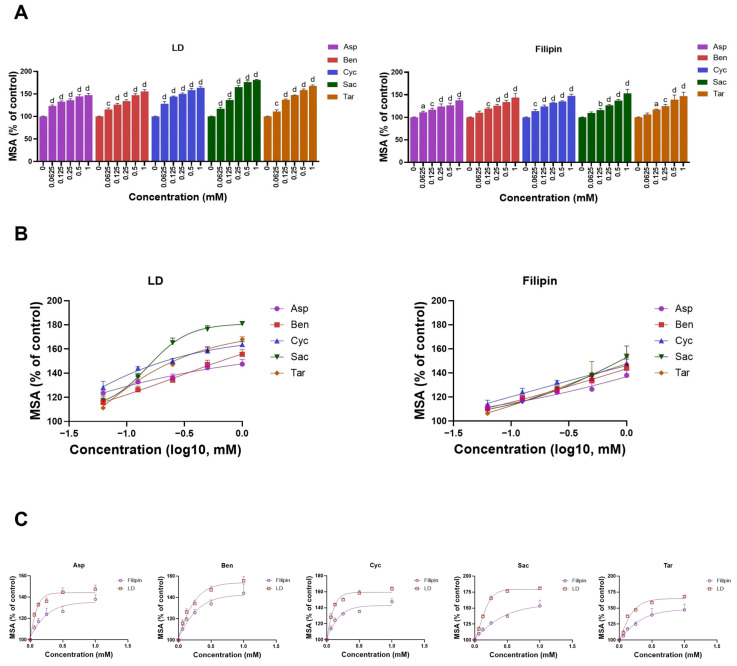
Effects of aspartame (Asp), sodium benzoate (Ben), cyclamate (Cyc), saccharin sodium (Sac) and tartrazine (Tar) on free cholesterol accumulation (Filipin) and lipid accumulation (LD) in Caco-2 cells. (**A**) The effect of five additives on the average dyeing area of Filipin and LD (histogram). (**B**,**C**) Dose-effect curves of five additives on Filipin and LD; Unpaired t test or one-way analysis of variance (one-way ANOVA) were used for significance test. a, *p* < 0.05; b, *p* < 0.01; c, *p* < 0.001; d, *p* < 0.0001; MSA (% of control): the percentage of the average staining area of the treatment group to the average staining area of the control group; The error bar represents the mean ± standard deviation of three biological replicates.

The same concentration gradient treatments (0–1 mM) were applied to Caco-2 cells, and no obvious cytotoxicity was observed at any concentration (Figure S1B). Consistent with the HepG2 model results, all food additives induced free cholesterol accumulation (Filipin staining, *p* < 0.001) and lipid accumulation (LD staining, *p* < 0.001) in Caco-2 cells in a dose-dependent manner (Figure 2A,B), with lipid accumulation showing a significantly greater increase than free cholesterol accumulation (Figure 2C). At low concentrations (≤0.0625 mM), aspartame, sodium benzoate, and sodium cyclamate exhibited stronger lipid accumulation-promoting effects than sodium saccharin and tartrazine; however, at high concentrations (≥0.5 mM), the effects of tartrazine and sodium saccharin became more pronounced (Figure 2B). Cell imaging analysis revealed that, at the 1 mM treatment concentration, all five food additives significantly increased MSA of free cholesterol (Filipin) and lipids (LD) compared with the control group (Figure S3). These findings demonstrate that the five food additives induce dose-dependent abnormal phenotypic alterations in cholesterol membrane localization and intracellular lipid deposition in Caco-2 cells, suggesting that food additive exposure can alter intestinal epithelial lipid-related phenotypes, which may serve as a potential risk factor for normal intestinal physiological function.

#### 3.1.3. Effects of Individual Food Additives on Lipid Metabolism in Jurkat T Cells

In non-activated Jurkat T cells (without PMA and ionomycin stimulation), treatment with the same concentration gradient showed no obvious cytotoxicity at any concentration (Figure S1C). The effects of the five food additives on lipid metabolism are presented in Figure S4. In non-activated Jurkat T cells, the five food additives did not trigger biologically relevant changes in intracellular free cholesterol (Filipin staining) or lipid droplet (LD staining) accumulation. Sporadic statistical differences were observed at isolated concentrations for some additives, but no monotonic dose–response relationship was detected in any group. For free cholesterol, significant differences were found at specific concentrations for all additives; tartrazine showed a slight upward trend without a continuous gradient. For lipid droplets, significant variations occurred at particular concentrations, and saccharin sodium exhibited a slight upward trend that peaked at 0.5 mM with no dose-dependent enhancement. Overall, the five additives produced only sporadic statistical differences and exerted no significant biological effects on cholesterol and lipid droplet accumulation. Cell imaging analysis revealed that, at the 1 mM treatment concentration, the Filipin staining areas of aspartame, sodium benzoate, sodium cyclamate, sodium saccharin, and tartrazine showed no significant differences compared with the control group, whereas only sodium benzoate- and sodium cyclamate-treated groups demonstrated an increasing trend in LD staining areas (Figure S5).

Jurkat T cells were pretreated with food additives for 48h, followed by activation with PMA and ionomycin for 12 h. The effects of the five food additives on lipid metabolism are presented in Figure S6; no cytotoxicity was observed in any treatment group (Figure S1D). Filipin and LD staining results showed that, within the concentration gradient of 0.0625–1 mM, sporadic statistical differences appeared at isolated concentrations for individual additives, and no monotonic dose–response relationship was observed across all treatments. For free cholesterol, significant differences occurred at specific concentrations for each additive, and tartrazine exhibited a slight downward trend without a continuous gradient; for lipid droplets, significant variations were also limited to discrete doses with no consistent dose-dependent enhancement.

### 3.2. Combined Effects of Food Additive Mixtures on Lipid Accumulation

Based on the dose–response relationship analysis of cholesterol accumulation (Filipin staining) and lipid accumulation (LD staining) induced by the five food additives in HepG2, Caco-2, and Jurkat T cells, lipid accumulation (LD) effects were significantly stronger than cholesterol accumulation (Filipin). In HepG2 and Caco-2 cells, both lipid metabolism indicators showed stable dose-dependent upward trends for all five food additives, whereas the five food additives had no significant effects on cholesterol accumulation (Filipin) or lipid accumulation (LD) in either resting or activated Jurkat T cells. Therefore, HepG2 and Caco-2 cells were selected for combined exposure experiments. To simulate low-concentration long-term exposure scenarios, BMD10 (benchmark dose at 10% effect level) was adopted as the core evaluation indicator according to EFSA guidelines. The BMD_10_ values and 95% confidence intervals (BMDL-BMDU) for Filipin and LD of the five food additives in both cell models were calculated (Table 1). The LD-BMD_10_ values were consistently lower than the Filipin-BMD_10_ values for all food additives; thus, lipid accumulation was selected as the endpoint for combined exposure assessment.

#### 3.2.1. Analysis of Combined Effects of Food Additives Based on HepG2 Cells

**Figure 3:**
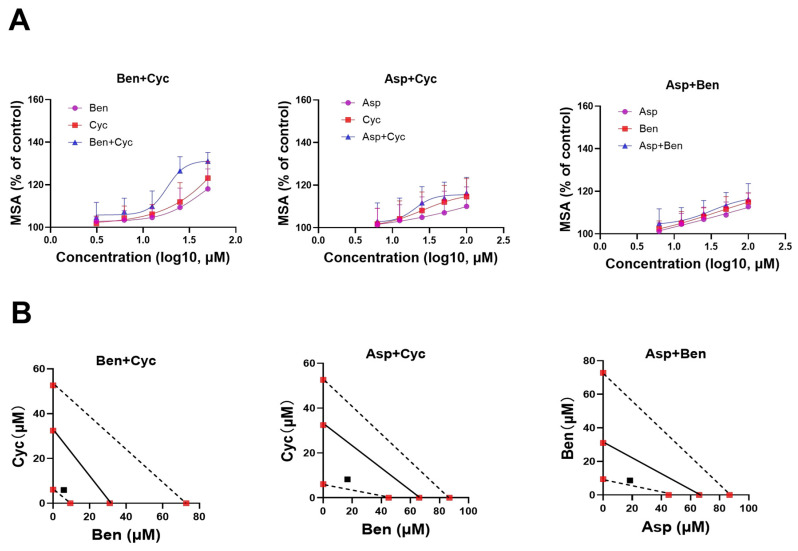
Effects of combined exposure of aspartame (Asp), sodium benzoate (Ben) and cyclamate (Cyc) at equal toxic ratios on lipid accumulation (LD) in HepG2 cells. (**A**) Effects of pairwise combinations on the mean stained area of LD in HepG2 cells. MSA (% of control): the percentage of the average staining area of the treatment group to the average staining area of the control group; the error bar represents the mean ± standard deviation of three biological replicates. (**B**) Isobologram analysis of pairwise combinations of two food additives. The dashed line connects the BMDL and BMDU of the two additives, and the solid line represents the connecting line of their BMD_10_ values. Black squares indicate the experimentally determined BMD_10_ of binary mixture treatments.

In HepG2 cells, the BMD_10_ values for cholesterol accumulation (Filipin) induced by aspartame, sodium benzoate, sodium cyclamate, sodium saccharin, and tartrazine were 91.07, 65.22, 77.59, 273.22, and 537.71 μM, respectively; the BMD_10_ values for lipid accumulation (LD) were 66.03, 31.04, 32.43, 135.00, and 312.00 μM, respectively (Table 1). Lower BMD_10_ values indicate higher cellular sensitivity to the corresponding effects induced by food additives. Therefore, the sensitivity ranking of HepG2 cells to the five food additives was identical for both cholesterol (Filipin) and lipid (LD) accumulation endpoints: sodium benzoate > sodium cyclamate > aspartame > sodium saccharin> tartrazine. Based on these results, the three food additives with the strongest effects (lowest BMD_10_ values)—sodium benzoate, sodium cyclamate, and aspartame—were selected for pairwise combined exposure experiments. Mixtures were prepared at equitoxic ratios (based on single-component BMD_10_ values) with concentration gradients. No cytotoxicity was observed in any treatment group (Figure S1E). The mixture LD-BMD_10_ values for all three combinations (sodium benzoate with sodium cyclamate, sodium benzoate with aspartame, and aspartame with sodium cyclamate) were lower than the corresponding single-component BMD_10_ values (Table S2). Dose–response curves (Figure 3A) and cell imaging analysis (Figures S7–S9) demonstrated enhanced lipid accumulation effects upon combined exposure. Isobologram analysis revealed that all mixture data points fell within the additivity domain (between the BMD_10_ line and BMDL line, Figure 3B), supporting an additive interaction. These findings indicate that sodium benzoate combined with sodium cyclamate, sodium benzoate combined with aspartame, and aspartame combined with sodium cyclamate exert additive effects on lipid accumulation.

#### 3.2.2. Analysis of Combined Effects of Food Additives Based on Caco-2 Cells

**Figure 4:**
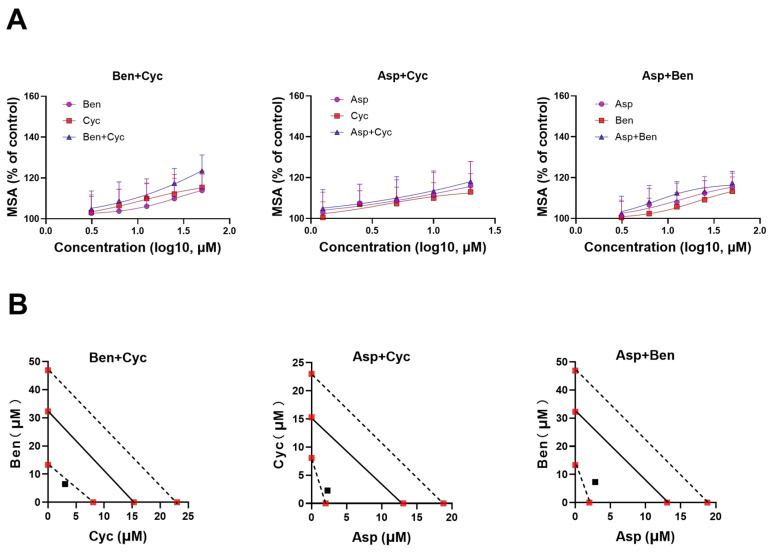
Effects of combined exposure of aspartame (Asp), sodium benzoate (Ben) and cyclamate (Cyc) at equal toxic ratios on lipid accumulation (LD) in Caco-2 cells. (**A**) Effects of pairwise combinations on the mean stained area of LD in Caco-2 cells. MSA (% of control): The percentage of the average staining area of the treatment group to the average staining area of the control group; the error bar represents the mean ± standard deviation of three biological replicates. (**B**) Isobologram analysis of pairwise combinations of two food additives. The dashed line connects the BMDL and BMDU of the two additives, and the solid line represents the connecting line of their BMD_10_ values. Black squares indicate the experimentally determined BMD_10_ of binary mixture treatments.

In Caco-2 cells, the BMD_10_ values for cholesterol accumulation (Filipin) induced by aspartame, sodium benzoate, sodium cyclamate, sodium saccharin, and tartrazine were 31.40, 51.92, 34.48, 60.39, and 77.96 μM, respectively; the BMD_10_ values for lipid accumulation (LD) were 13.14, 32.26, 15.30, 41.98, and 47.54 μM, respectively (Table 1). The sensitivity ranking was identical for both endpoints: aspartame > sodium cyclamate > sodium benzoate > sodium saccharin> tartrazine. Notably, this ranking differed markedly from that observed in HepG2 cells (Table 1), suggesting that the disruptive effects of food additives on lipid metabolism are cell type-specific, possibly attributable to differences in metabolic pathways, transporter expression, or staining characteristics between cell lines. The three food additives with the strongest effects (lowest BMD_10_ values)—aspartame, sodium cyclamate, and sodium benzoate—were selected for pairwise combined exposure experiments. Mixtures were prepared at equitoxic ratios (based on single-component BMD_10_ values) with concentration gradients. No cytotoxicity was observed in any treatment group (Figure S1F). The mixture LD-BMD_10_ values for all three combinations (aspartame with sodium cyclamate, aspartame with sodium benzoate, and sodium benzoate with sodium cyclamate) were lower than the corresponding single-component BMD_10_ values (Table S3). Dose–response curves (Figure 4A) and cell imaging analysis (Figures S10–S12) demonstrated enhanced lipid accumulation effects upon combined exposure. Isobologram analysis revealed that data points for aspartame combined with sodium cyclamate and aspartame combined with sodium benzoate fell within the additivity domain (between the BMD_10_ line and BMDL line), supporting additive interactions; whereas data points for sodium benzoate combined with sodium cyclamate fell within the synergism domain (below the BMDL line), supporting a synergistic interaction (Figure 4B). These findings indicate that aspartame combined with sodium cyclamate, aspartame combined with sodium benzoate, and sodium benzoate combined with sodium cyclamate exert additive, additive, and synergistic effects on lipid accumulation, respectively.

## 4. Discussion

### 4.1. Sensitivity of Phenotypic Markers for Lipid Metabolism

Potential perturbations in lipid metabolic homeostasis represent a critical manifestation of potential health risks associated with food additives, and biomarker sensitivity directly determines the precision of risk assessment. We demonstrated that in HepG2 and Caco-2 cells, lipid droplet (LD) accumulation exhibited significantly greater sensitivity to five food additives compared to free cholesterol accumulation (Filipin staining), as evidenced by lower BMD_10_ values for LD induction across all additives. This phenotypic divergence stems from distinct metabolic regulatory characteristics: LDs, as core storage depots for neutral lipids such as triglycerides, possess highly flexible biogenesis and metabolic dynamics, enabling rapid accumulation in response to altered lipid supply or turnover [48,49,50]. In contrast, intracellular cholesterol distribution is strictly buffered by robust homeostatic mechanisms, resulting in a delayed response to perturbation as observed here [51]. This inherent biological difference underscores the superior sensitivity of LD accumulation as an early phenotypic indicator of lipid metabolism disturbance induced by food additives [52,53].

### 4.2. Cell-Specific Lipid Metabolic Responses to Food Additives and Underlying Mechanisms

The functional specificity of lipid metabolism between metabolic cells (hepatocytes and intestinal epithelial cells) and immune cells determines their differential responses to food additives. We observed no dose-dependent lipid metabolic regulation in Jurkat T cells, with none of the five additives significantly affecting Filipin or LD accumulation in resting or activated states—a striking contrast to the persistent lipid-related phenotypic abnormalities observed in HepG2 and Caco-2 cells. This profound cell-type specificity highlights that metabolic vulnerability to food additives is intrinsically linked to cellular function. This divergence reflects distinct lipid metabolic priorities: metabolic cells are specialized for lipid processing and storage, whereas immune cells like T cells utilize lipids primarily for signaling and membrane dynamics [54,55]; altered lipid metabolism, a characteristic feature of metabolic liver dysfunction, has been reported to correlate with tissue microenvironment remodeling and immune cell functional regulation in a prior study [56].

This cell-specific divergence can be further elaborated by the distinct biological roles of each cell type, as supported by recent studies. For HepG2 hepatocytes (a classic hepatic model), exogenous chemical-induced lipid accumulation in HepG2 cells has been previously correlated with hepatic injury-related phenotypes including mitochondrial dysfunction and oxidative stress in published research [57]. Caco-2 cells exhibit active lipid transport capabilities [58]. In sharp contrast, Jurkat T cells, as immune cells, undergo metabolic reprogramming upon activation that prioritizes cellular functions distinct from bulk lipid storage [59]. Unlike metabolic cells, where LDs primarily serve as energy storage organelles, LDs in T cells are involved in different cellular processes, and such inherent functional differences across cell types provide a reasonable contextual explanation for the distinct lipid phenotypic responses observed in the present study [59].

The inconsistent sensitivity ranking of the five food additives between HepG2 and Caco-2 cells can be attributed to their inherent lineage-specific lipid metabolic frameworks. These intrinsic differences may collectively lead to divergent response magnitudes and inconsistent potency rankings when exposed to the same panel of food additives. Combined exposure tests were not performed in Jurkat T cells in the current study, which should be explicitly acknowledged as a study limitation. The primary rationale lies in the absence of any significant, dose-dependent lipid phenotypic changes induced by individual additives in Jurkat cells, precluding meaningful assessment of combinatorial effects on these specific endpoints. Therefore, further validation using models exhibiting baseline susceptibility would be required in future work to explore potential immunometabolic interactions under co-exposure.

These findings indicate that food additive metabolic effects exhibit marked cell specificity linked to functional status, suggesting that cell functional status must be incorporated as a critical covariate in safety assessment to avoid underestimating potential risks under specific physiological scenarios (e.g., infection, inflammation).

Current food safety risk assessment frameworks remain predominantly focused on individual chemicals, establishing health guidance values such as Acceptable Daily Intakes (ADI) or employing Margin of Exposure (MOE) approaches. These single-chemical models fail to effectively reveal potential interactive effects—including synergism or antagonism—among additives, leading to underestimation of real-world mixture exposure risks [60,61]. Consequently, international regulatory bodies including the Joint FAO/WHO Expert Committee on Food Additives (JECFA), the European Food Safety Authority (EFSA), and the U.S. Environmental Protection Agency (EPA) are accelerating the transformation of risk assessment paradigms to incorporate chemical interactions into cumulative exposure assessment frameworks [62,63,64].

### 4.3. Cell-Specific Interactions Under Combined Exposure

Through pairwise combined exposure experiments, we revealed cell-specific interaction patterns that underscore this regulatory urgency. In HepG2 cells, sodium benzoate-sodium cyclamate, aspartame-sodium benzoate, and aspartame-sodium cyclamate combinations all exhibited additive effects, as demonstrated by lower LD-BMD_10_ values and isobologram data points distributed within the additivity domain (between BMDL and BMD_10_ lines), suggesting these additives induce lipid phenotypic alterations via non-synergistic, independent functional pathways under our experimental conditions. In Caco-2 cells, aspartame-sodium cyclamate and aspartame-sodium benzoate remained additive, whereas the sodium benzoate-sodium cyclamate combination showed synergistic effects with data points below the BMDL line. This synergistic interaction in intestinal epithelial cells is of high relevance for risk assessment: synergism indicates that the combined effect exceeds the sum of individual effects, suggesting that even low-dose co-exposure to these two additives may trigger abnormal lipid-related phenotypes unanticipated by single-additive toxicity data. This striking cell-specific divergence is consistent with the distinct physiological functions of intestinal and hepatic models: Caco-2 cells serve as the primary intestinal exposure and first-pass metabolic interface with stronger responsiveness to exogenous chemical co-exposure, while HepG2 hepatocytes exert stronger lipid homeostatic buffering, as widely reported in gut-liver axis models [58,65].

Biologically, the predominant additive interactions observed in HepG2 cells under our experimental conditions suggest that hepatic lipid responses to these specific pairwise combinations may follow a dose-additive paradigm, consistent with the strong homeostatic regulatory capacity of hepatocytes. While this observation provides preliminary support for the potential applicability of dose-addition principles in this context, it is critical to emphasize that our in vitro findings cannot be directly translated into regulatory guidance. Further validation using more complex mixture scenarios, primary cell models, and in vivo systems is required before any extrapolation to humans.

### 4.4. Limitations of the Present Study

Notably, the present HCI data only reflect alterations in lipid droplet accumulation and free cholesterol staining patterns, representing lipid-related phenotypic changes rather than direct evidence of variations in lipid metabolic flux, de novo synthesis, oxidation, or transport. Meanwhile, such interaction effects were particularly pronounced at BMD_10_-equivalent doses, highlighting the limitations of current risk assessment relying on single-component ADIs, which fails to cover multi-component synergistic effects and may underestimate metabolic risks under real-world dietary scenarios. BODIPY 493/503 was used in this study to label neutral lipids (mainly triglycerides and cholesterol esters) in lipid droplets, while Filipin complex specifically stains cellular free cholesterol, whereas Filipin complex was applied to detect cellular free cholesterol. It should be noted that lipid droplets naturally harbor various neutral lipid components beyond these categories. Consequently, the present study only implemented overall phenotypic quantification for preliminary screening of lipid metabolic disruption, without carrying out detailed identification or compositional analysis of the internal lipid constituents.

Furthermore, several inherent limitations of this study should be acknowledged. First, all three cell models (HepG2, Caco-2, and Jurkat T cells) are immortalized transformed cell lines rather than primary human cells or intact physiological tissues. As such, the current in vitro findings cannot be directly extrapolated to native human physiological conditions, and further validation using primary cells or in vivo models remains indispensable. Second, the present investigation only covers a limited scope of food additive combinations and concentration scenarios. Taken together, considerable caution is warranted when extending these in vitro phenotypic observations—particularly the preliminary dose-addition findings in HepG2 cells—to systematic toxicological explanations or broader food safety regulatory implications. No definitive conclusions regarding regulatory extrapolation can be drawn from the present in vitro data alone.

## 5. Conclusions

In summary, this multi-cellular model study demonstrates that combined exposure to multiple food additives, including aspartame, sodium benzoate, sodium cyclamate, sodium saccharin, and tartrazine, can induce obvious lipid-related abnormal cellular phenotypes in HepG2 and Caco-2 cell lines. Within the specific experimental conditions, endpoints, and cell systems evaluated herein, lipid droplet (LD) accumulation served as a more sensitive phenotypic indicator than free cholesterol staining for detecting chemical-induced lipid-associated phenotypic anomalies, supporting its potential utility for in vitro screening in food additive safety assessment. Pairwise combinations revealed cell-specific interaction patterns: additive effects predominated in HepG2 cells, whereas synergistic effects for sodium benzoate-sodium cyclamate were observed in Caco-2 cells. These findings underscore that combined exposure to food additives at the tested doses induces distinct lipid-related cellular phenotypic changes not captured by current single-component risk assessment frameworks, highlighting the critical need to incorporate mixture toxicity evaluation into future food safety research and regulatory development.

## Figures and Tables

**Table 1:** BMD_10_ values and 95% confidence intervals of five additives on Filipin and LD in HepG2 cells and Caco-2 (μM).

Fluorescent Probe	Food Additive	BMD_10_ (BMDL-BMDU)	
		HepG2 Cells	Caco-2 Cells
Filipin	Aspartame	91.07 (30.00–170.00)	31.40 (12.40–106.00)
	Sodium Benzoate	65.22 (34.20–122.00)	51.92 (13.60–93.60)
	Cyclamic acid sodium	77.59 (33.30–179.00)	34.48 (7.86–53.30)
	Saccharin Sodium	273.22 (154.00–436.00)	60.39 (28.30–99.10)
	Tartrazine	537.71 (410.00–695.00)	77.96 (20.60–141.00)
LD	Aspartame	66.03 (45.00–86.90)	13.14 (2.00–18.80)
	Sodium Benzoate	31.04 (9.40–72.80)	32.26 (13.30–46.90)
	Cyclamic acid sodium	32.43 (10.00–52.60)	15.30 (8.08–23.00)
	Saccharin Sodium	135.00 (51.00–292.00)	41.98 (34.70–51.50)
	Tartrazine	312.00 (140.00–599.00)	47.54 (34.00–59.20)

## Data Availability

The original contributions presented in this study are included in the article/Supplementary Materials. Further inquiries can be directed to the corresponding authors.

## Supplementary Materials

The following supporting information can be downloaded at: https://www.mdpi.com/article/10.3390/toxics14060487/s1, Table S1: Reagents and key resources; Table S2: BMD_10_ values and 95 % confidence intervals for LD in HepG2 cells (μM) exposed to three additives alone and in pairwise combinations; Table S3: BMD_10_ values and 95 % confidence intervals for LD in Caco-2 cells (μM) exposed to three additives alone and in pairwise combinations; Figure S1: Effect of test substance on cell number in various cell models; Figure S2: Representative images of Filipin and LD staining after treatment of HepG2 cells with five food additives (1 mM); Figure S3: Representative images of Filipin and LD staining after treatment of Caco-2 cells with five food additives (1 mM); Figure S4: Effects of aspartame (Asp), sodium benzoate (Ben), cyclamate (Cyc), saccharin sodium (Sac) and tartrazine (Tar) on free cholesterol accumulation (Filipin) and lipid accumulation (LD) in Jurkat T; Figure S5: Representative images of Filipin and LD staining after Jurkat T cells were treated with five additives (1 mM); Figure S6: Effects of aspartame (Asp), sodium benzoate (Ben), cyclamate (Cyc), saccharin sodium (Sac) and tartrazine (Tar) on free cholesterol accumulation (Filipin) and lipid accumulation level (LD) in activated Jurkat T cells; Figure S7: Representative images of Filipin and LD staining in HepG2 cells following combined treatment with sodium benzoate (Ben) and sodium cyclamate (Cyc); Figure S8: Representative images of Filipin and LD staining in HepG2 cells following combined treatment with aspartame (Asp) and sodium cyclamate (Cyc); Figure S9: Representative images of Filipin and LD staining in HepG2 cells following combined treatment with aspartame (Asp) and sodium benzoate (Ben); Figure S10: Representative images of Filipin and LD staining in Caco-2 cells following combined treatment with sodium benzoate (Ben) and sodium cyclamate (Cyc); Figure S11: Representative images of Filipin and LD staining in Caco-2 cells following combined treatment with aspartame (Asp) and sodium cyclamate (Cyc); Figure S12: Representative images of Filipin and LD staining in Caco-2 cells following combined treatment with aspartame (Asp) and sodium benzoate (Ben).

## Abbreviations

The following abbreviations are used in this manuscript:

ADI	Acceptable Daily Intake
ANOVA	Analysis of variance
Asp	Aspartame
ATCC	American type culture collection
Ben	Sodium benzoate
BMD	Benchmark Dose
BMR	Benchmark Response
Cyc	Sodium cyclamate
DMSO	Dimethyl sulfoxide
EPA	Environmental Protection Agency
EFSA	European Food Safety Authority
FBS	Fetal bovine serum
HCI	Live-cell high content imaging
IARC	International Agency for Research on Cancer
JECFA	Joint FAO/WHO Expert Committee on Food Additives
LD	Lipid droplet
LDL	Low-Density Lipoprotein
MSA	Mean stain area
NAMs	New Approach Methodologies
PBS	Phosphate-buffered solution
PMA	Phorbol 12-myristate 13-acetate
P/S	Penicillin/Streptomycin
PPARγ	Peroxisome proliferator-activated receptor gamma
Sac	Sodium saccharin
SREBP	Sterol Regulatory Element-Binding Protein
Tar	Tartrazine
TG	Triglycerides
TGF-β	Transforming Growth Factor-β
7AAD	7-Aminoactinomycin D
