# Supplementary material for: Lipid Metabolic Effects Induced by Individual and Combined Exposure to Multiple Food Additives in Human Cells
Source: Toxics. 2026 Jun 3;14(6):487. doi: 10.3390/toxics14060487 (PMC13307439; doi:10.3390/toxics14060487)
Supplement: Supplementary file 1 [file toxics-14-00487-s001.zip › toxics-4268548-supplementary.pdf]

Table S1. Reagents and key resources.

REAGENT or RESOURCE	SOURCE	IDENTIFIER
Compounds		
Aspartame	Sigma-Aldrich	Cat#PHR1381<br>CAS#22839-47-0
Sodium benzoate	Sigma-Aldrich	Cat#PHR1231<br>CAS#532-32-1
Cyclamic acid sodium	Aladdin Biochemical Technology Co., Ltd.	Cat#S109885<br>CAS#139-05-9
Saccharin sodium	Sigma-Aldrich	Cat#PHR1348<br>CAS#128-44-9
Tartrazine	Aladdin Biochemical Technology Co., Ltd.	Cat#T102072<br>CAS#1934-21-0
Critical Commercial Assays		
Roswell Park Memorial Institute (RPMI) 1640	Thermo Scientific	Cat#11875093
Fetal Bovine Serum (FBS)	Thermo Scientific	Cat#10270106
Penicillin/Streptomycin(P/S)	Thermo Scientific	Cat#15140122
4% paraformaldehyde fixative	Solarbio	Cat#IP9321
Dulbecco's Modified Eagle Medium	Thermo Scientific	Cat#11995065
Trypsin-EDTA (0.25%), phenol red	Thermo Scientific	Cat#25200056
Eagle's Minimum Essential Medium (EMEM）	American Type Culture Collection	Cat#30-2003
Accutase	Stemcell Technologies	Cat#7920
DMSO	Sigma-Aldrich	Cat#D2438-5X10ML
PMA	Sigma-Aldrich	Cat#P1585-1MG
Filipin complex	MCE	Cat#HY-N6716
BODIPY 493/503（LD）	Bioss	Cat#HY-W090090
7-AAD Viability Staining Solution	BD PMG	Cat#559925
PMA	Sigma-Aldrich	Cat#P8139-1mg
Ionomycin calcium salt	MCE	Cat#HY-13434A
Experimental Models: Cell Lines		
Jurkat T	American Type Culture Collection	Cat#TIB-152;
HepG2	Cell Bank of Type Culture Collection Committee of the Chinese Academy of Sciences	Cat#SCSP-510;
Caco-2	Cell Resource Center，Institute of Basic Medical Sciences, CAMS/PUMC	Cat#1101HUM-PUMC000100;

Software and Algorithms		
GraphPad Prism	GraphPad Software (version 10.0)	https://www.graphpad.com/
ImageXpress(v6.0)	Molecular Device	https://www.moleculardevices.com
European Food Safety Authority web tool based on Rpackage PROAST version 70.0	Shi et al., 2020	https://efsab2c.b2clogin.com/

Table S2 BMD_{10} values and 95 % confidence intervals for LD in HepG2 cells (μM) exposed to three additives alone and in pairwise combinations

**Food additive**	**BMD_{10}**	**BMDL**	**BMDU**
Aspartame	66.03	45.00	86.90
Sodium Benzoate	31.04	9.40	72.80
Cyclamic acid sodium	32.43	10.00	52.60
Sodium Benzoate + Cyclamic acid sodium	11.81	4.92	27.50
Aspartame + Cyclamic acid sodium	24.93	9.43	56.40
Aspartame + Sodium Benzoate	27.01	20.00	47.10

Table S3 BMD_{10} values and 95 % confidence intervals for LD in Caco-2 cells (μM) exposed to three additives alone and in pairwise combinations

**Food additive**	**BMD_{10}**	**BMDL**	**BMDU**
Aspartame	13.14	2.00	18.80
Sodium Benzoate	32.26	13.30	46.90
Cyclamic acid sodium	15.30	8.08	23.00
Sodium Benzoate + Cyclamic acid sodium	4.59	4.08	5.24
Aspartame + Cyclamic acid sodium	10.15	3.25	25.8
Aspartame + Sodium Benzoate	9.51	6.17	17.5

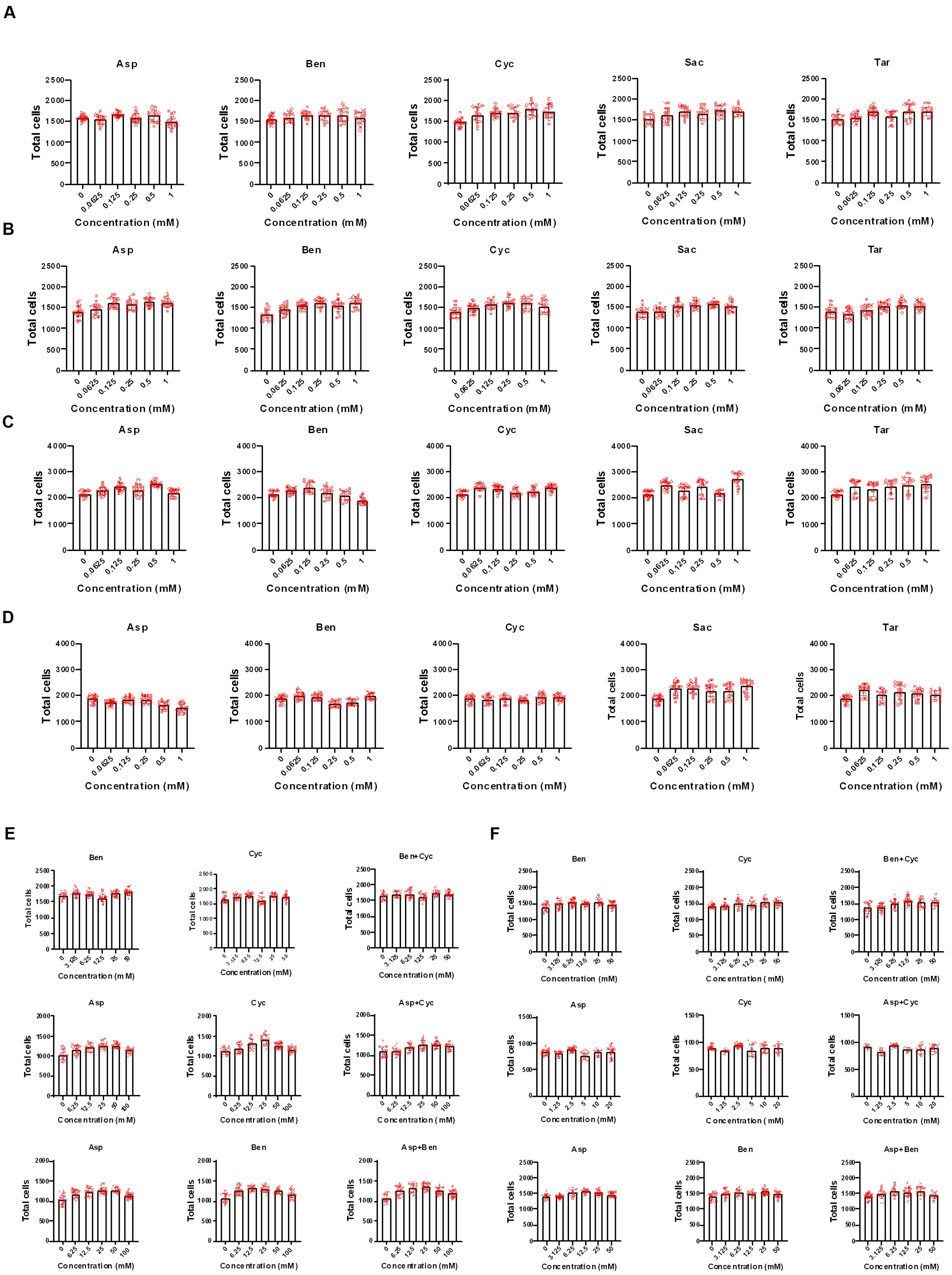

Figure S1. Effect of test substance on cell number in various cell models. (A) Cell number of HepG2 cells; (B) Cell number of Caco-2 cells; (C) Cell number of unactivated Jurkat T cells.;(D) Cell number of activated Jurkat T cells; (E) The number of HepG2 cells in the combined test; (F) The number of Caco-2 cells in the combined test.

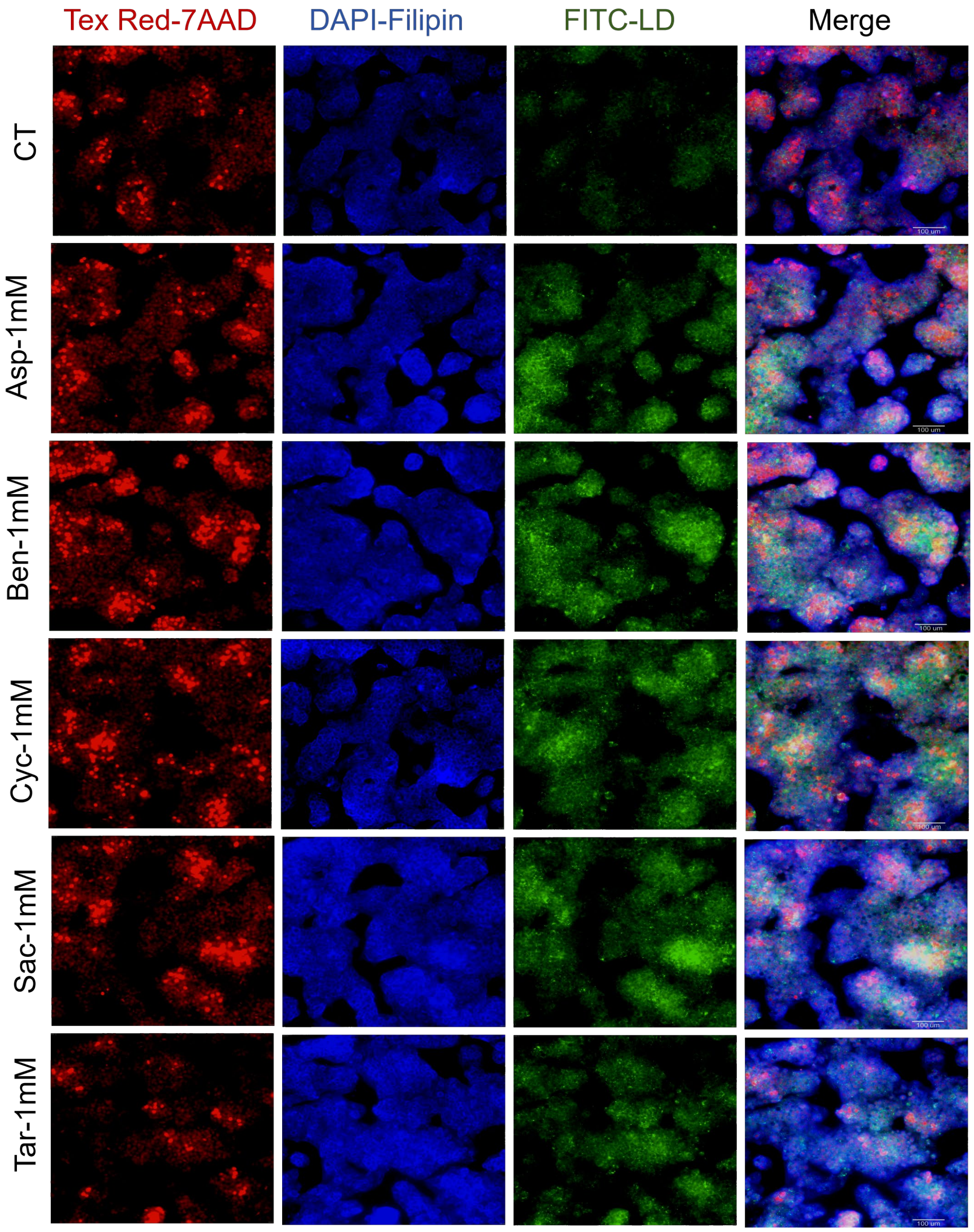

Figure S2. Representative images of Filipin and LD staining after treatment of HepG2 cells with five food additives (1 mM).

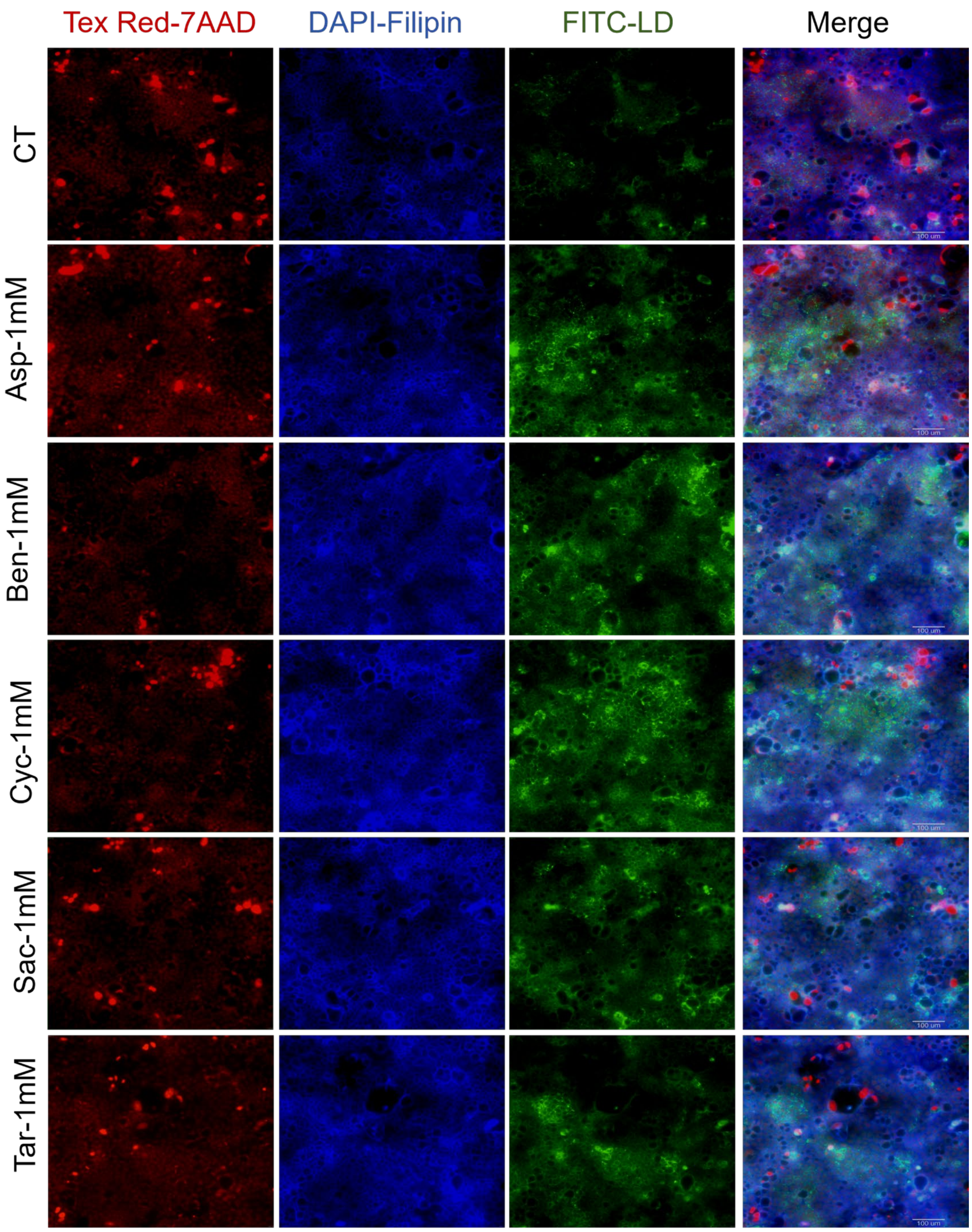

Figure S3. Representative images of Filipin and LD staining after treatment of Caco-2 cells with five food additives (1 mM).

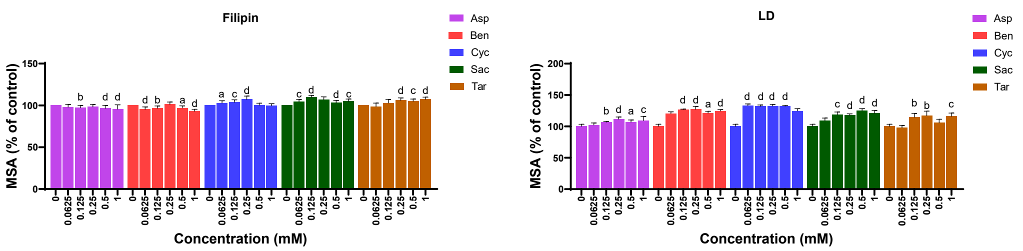

Figure S4. Effects of aspartame (Asp), sodium benzoate (Ben), cyclamate (Cyc), saccharin sodium (Sac) and tartrazine (Tar) on free cholesterol accumulation (Filipin) and lipid accumulation (LD) in Jurkat T. T' test or Kruskal-wallis test was used for significance test. a, $P < 0.05$; b, $P < 0.01$; c, $P < 0.001$; d, $P < 0.0001$; MSA (% of control) : the percentage of the average staining area of the treatment group to the average staining area of the control group ; The error bar represents the mean±standard deviation of three biological replicates.

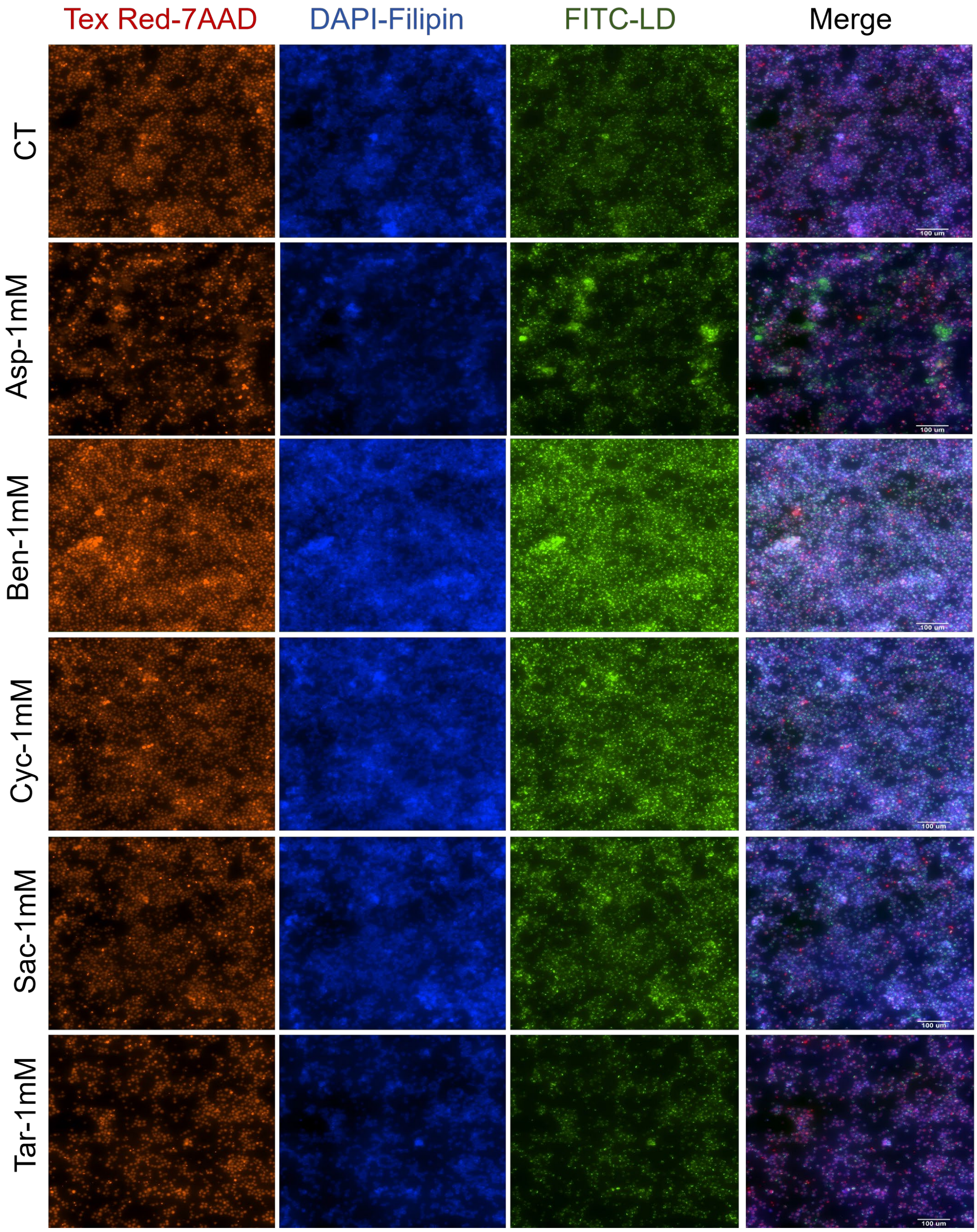

Figure S5. Representative images of Filipin and LD staining after Jurkat T cells were treated with five additives (1 mM).

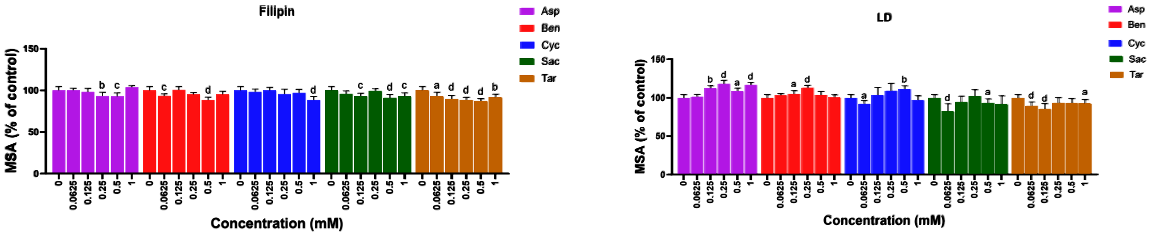

Figure S6. Effects of aspartame (Asp), sodium benzoate (Ben), cyclamate (Cyc), saccharin sodium (Sac) and tartrazine (Tar) on free cholesterol accumulation (Filipin) and lipid accumulation level (LD) in activated Jurkat T cells.The effect of five additives on the average staining area of Filipin and LD in Jurkat T cells after pretreatment and activation ; T' test or Kruskal-wallis test was used for significance test. a, $P < 0.05$; b, $P < 0.01$; c, $P < 0.001$; d, $P < 0.0001$; MSA (% of control) : the percentage of the average staining area of the treatment group to the average staining area of the control group ; the error bar represents the mean±standard deviation of the three biological replicates.

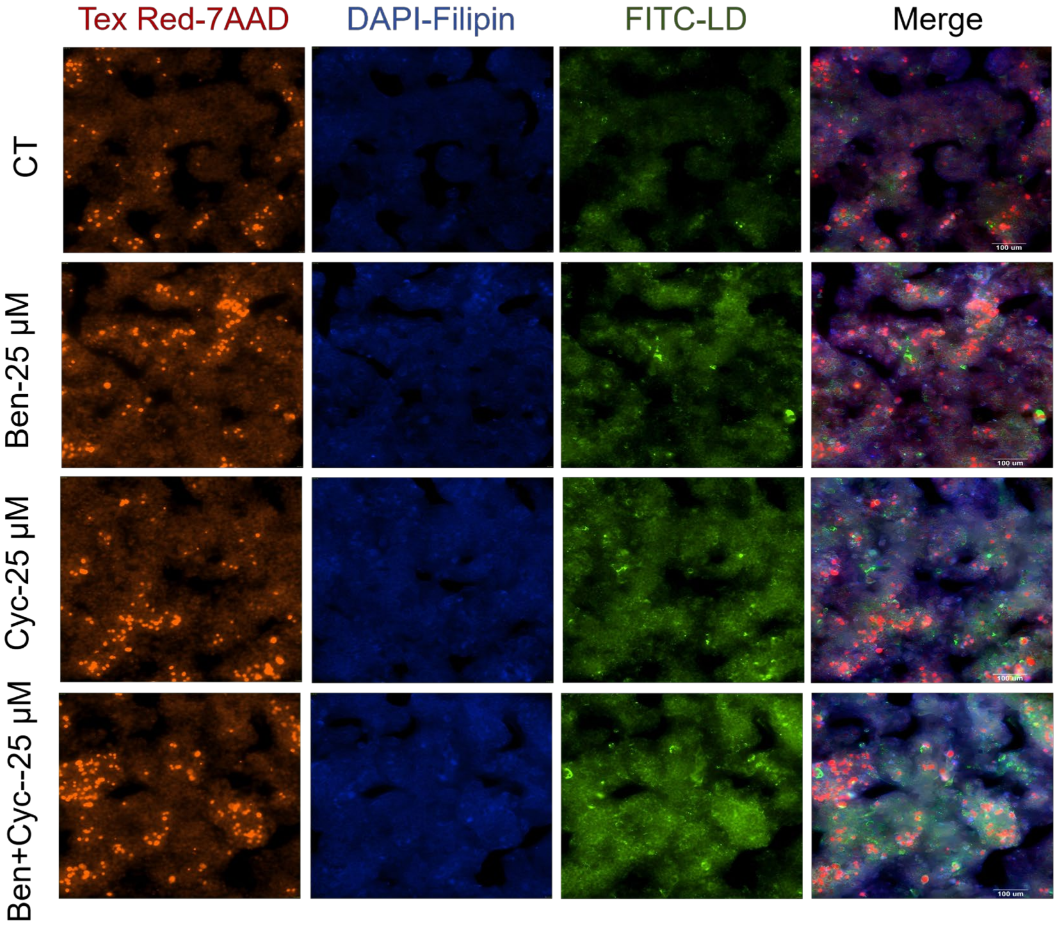

Figure S7. Representative images of Filipin and LD staining in HepG2 cells following combined treatment with sodium benzoate (Ben) and sodium cyclamate (Cyc).

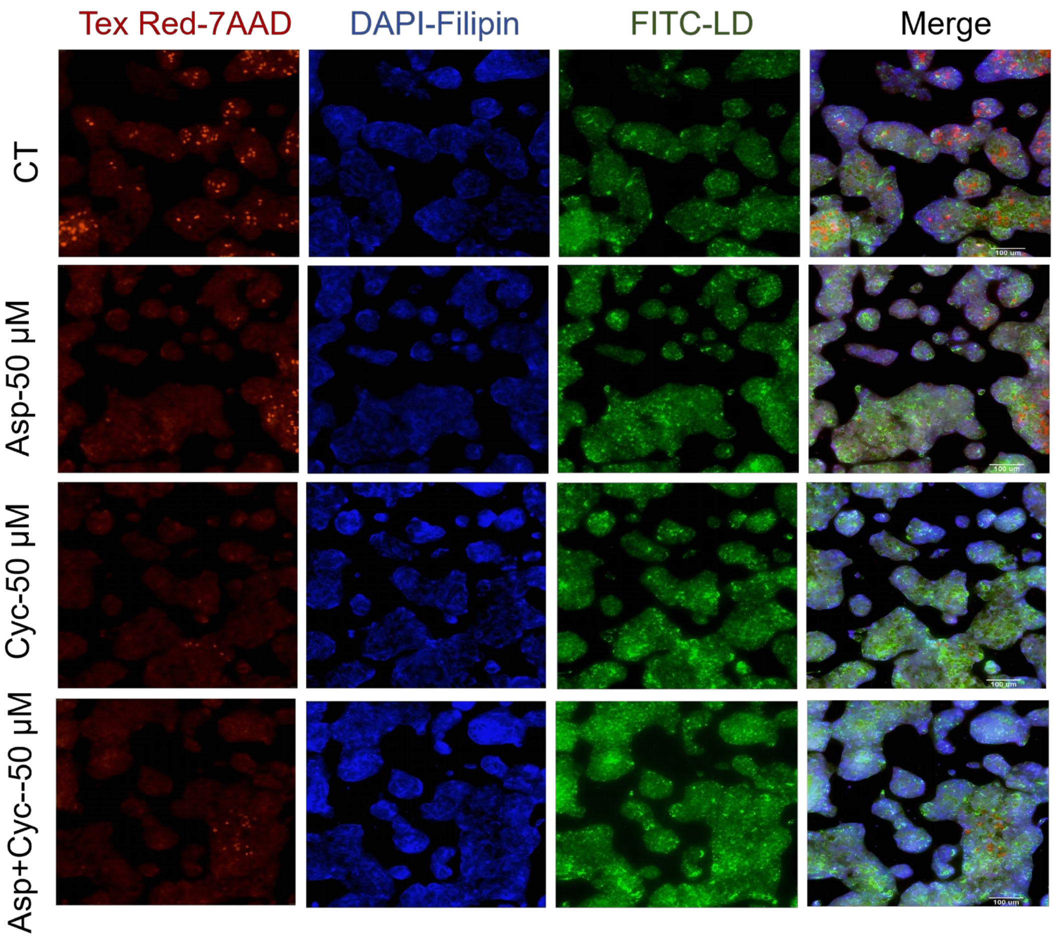

Figure S8. Representative images of Filipin and LD staining in HepG2 cells following combined treatment with aspartame (Asp) and sodium cyclamate (Cyc). The CT and Asp-50 μM groups are shared controls from the same cell plate as Figure S9.

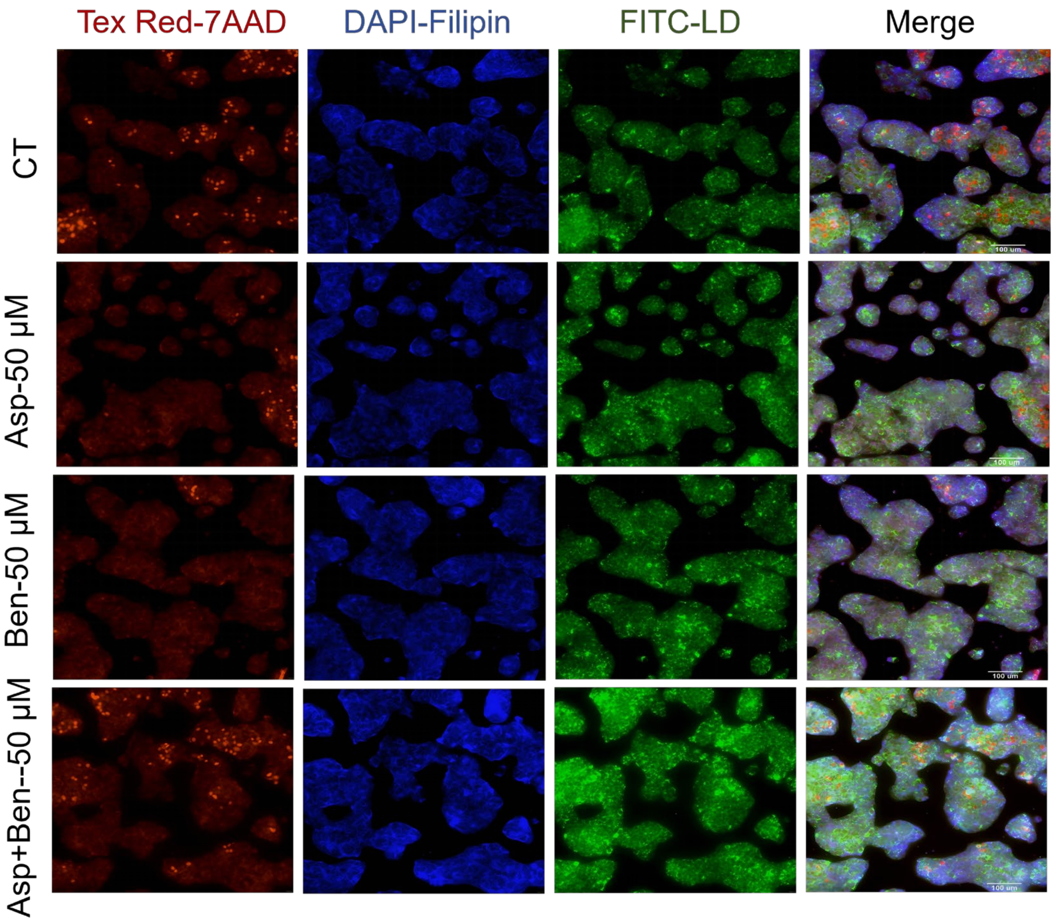

Figure S9. Representative images of Filipin and LD staining in HepG2 cells following combined treatment with aspartame (Asp) and sodium benzoate (Ben). The CT and Asp-50 μM groups are shared controls from the same cell plate as Figure S8.

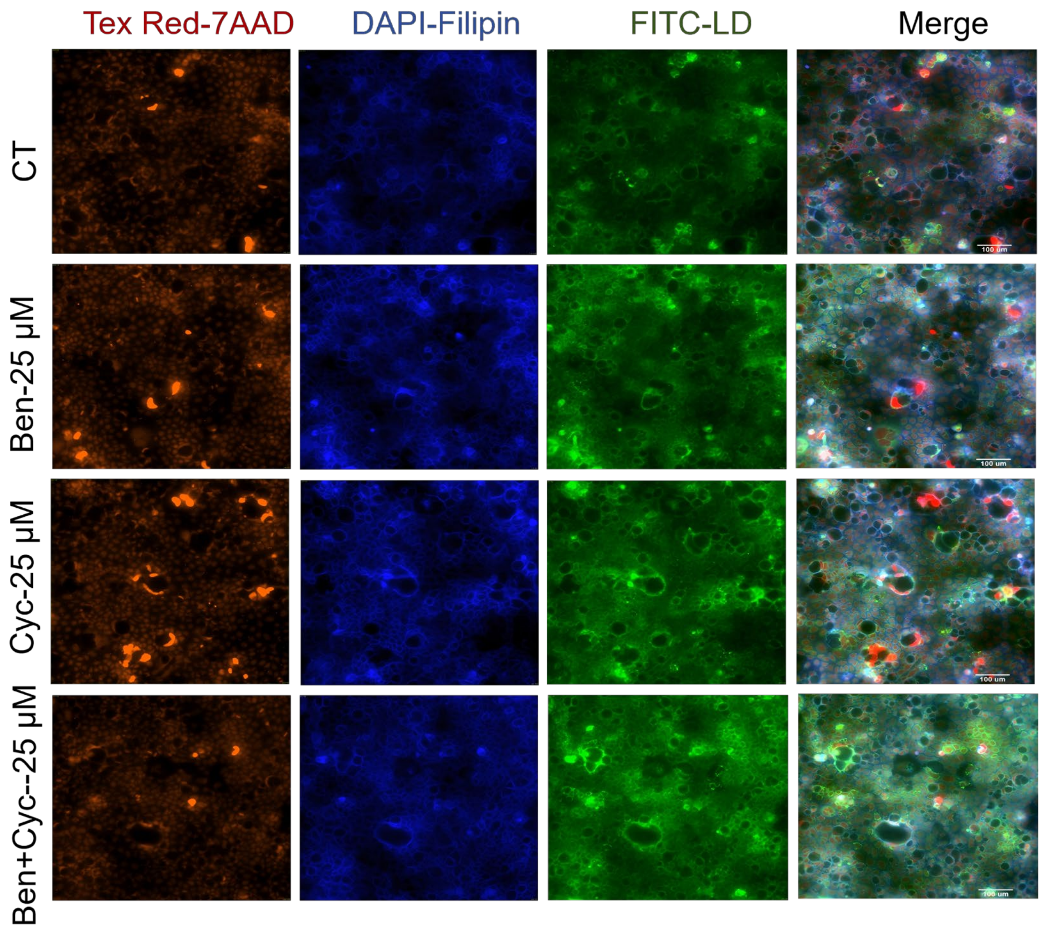

Figure S10. Representative images of Filipin and LD staining in Caco-2 cells following combined treatment with sodium benzoate (Ben) and sodium cyclamate (Cyc). The CT and Ben-25 μM groups are shared controls from the same cell plate as Figure S12.

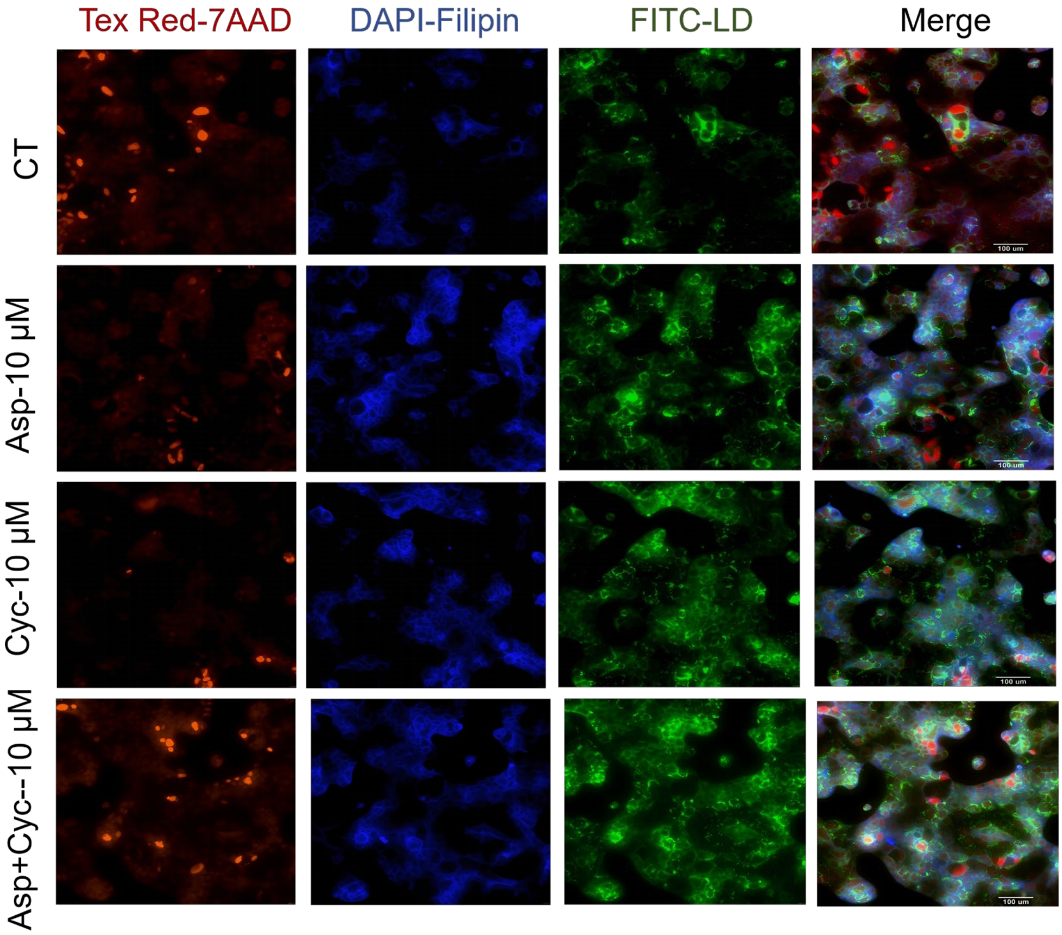

Figure S11. Representative images of Filipin and LD staining in Caco-2 cells following combined treatment with aspartame (Asp) and sodium cyclamate (Cyc).

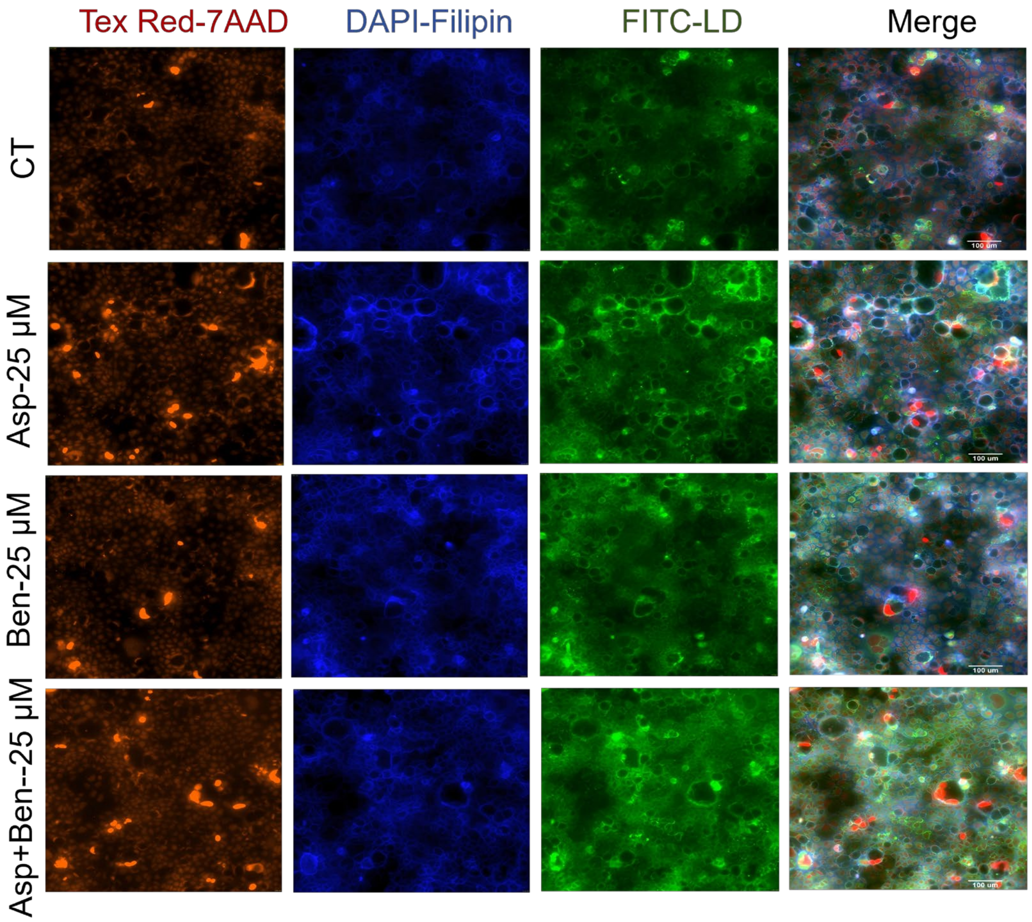

Figure S12. Representative images of Filipin and LD staining in Caco-2 cells following combined treatment with aspartame (Asp) and sodium benzoate (Ben). The CT and Ben-25 μM groups are shared controls from the same cell plate as Figure S10.
